# Temperamental Selection of Institutional Patients

**Published:** 1920-01-03

**Authors:** 


### TEMPERAMENTAL SELECTION OF INSTITUTIONAL PATIENTS.

In selecting patients for certain courses of treatment at institutes and "colonies" the temperamental as well as the medical suitability of the applicant should be taken into consideration. That is a point usually ignored, and yet often of the utmost importance. Prolonged institutional or "colonial" treatment necessarily involves a rather rigid and restricted environment, to which patients of certain temperaments and characters can never adapt themselves, and, if these unadaptable subjects are accepted for treatment, it means constant grousing, grumbling, fretting, and friction. The "unadaptables" do not themselves settle down, and by their discontent they unsettle those who might have done so. A single fretful patient, with a peevish tongue, can infect a whole peaceful community with the germ of discontent, and the germ, once sown, is difficult to eradicate.

It may not be at all easy to define the temperament and character suited for prolonged "colonial" or institutional treatment, but a doctor with natural discernment or experienced in the management of men should be able to select rightly in ninety-eight cases out of a hundred. It is not so much docility and tractability that are requisite as a combination of social instincts and individual resource. On the one hand, there is a rubbing of shoulders and a rubbing of characters, and with some people such rubbing necessarily implies friction. A man naturally unsociable, selfish, with no human sympathies, with no capacity to give and take, will find compulsory intercourse with others a source of constant irritation. Or even if he be fairly sociable, unselfish, and sympathetic, he may find promiscuous propinquity trying and intolerable. On the other hand, institutional and "colonial" *régime* must leave lacunæ in most men's lives which only personal resources can fill. A man whose character has crystallised or fossilised in a limited local environment, who has been for decades a puppet of routine, and who has no hobbies and no resources in himself, will never be able to adapt himself comfortably to institutional and "colonial" life. The unsocial man and the unresourceful man, and the man at once unsocial and unresourceful, will be unhappy and will "grouse," and will not only invent grievances or magnify grievances to explain their unhappiness and their grousing, but will encourage others to find grievances too.

In the selection of nerve cases due consideration of the temperament and character of the patient is especially desirable, for when the nerves are loose and slack temperament and character are often the last reins which can make a man persevere either in treatment or in work.

Patients, moreover, must be not only selected, but care must be taken that the selected cases do not deteriorate under prolonged treatment. It must be recognised that even social and resourceful, adaptable and contented patients tend to lose initiative, ambition, and backbone if they stay for any length of time under sheltered and easy conditions. They may regain physical health, but it is at the cost of volitional vigour. Every doctor who has had much experience of institutions must have seen patients who have been robbed of all industry and enterprise by institutional life, and who have become "slackers," patients whose one ambition is to find a "cushy" job requiring neither mental nor physical effort. Every doctor with sanatorium experience must know patients in whom "lazing" has become an incurable habit, and whose sole interests in life are their sputa, temperatures, and pulses.

The temperamental selection of patients is a matter, as we have said, of great importance, and the selection must be made by the doctor who is to have charge of them. But it must not be forgotten that the character and temperament of the doctor and of the nursing staff are as important as the character and temperament of the patients, and that both doctor and staff must be chosen as much on grounds of personal character as of professional ability. That, too, is a point that cannot be too much emphasised, and that is often entirely neglected. A doctor may be a man of skill, learning, and experience, and yet, without the personal qualities required for influencing and inspiring patients, may be a complete failure as head of an institution. A nurse or matron may be conscientious, industrious, well trained, and yet be a constant source of disharmony and discontent. The doctor's personality it is, in the first place, that must give moral and intellectual tone to the little community he rules; his personality it is that must inspire the spirit of the relationship between the patients and the spirit with which the patients combat disease and face life and death. If the doctor's morals, courage, and enthusiasm sink, it will affect every member of the community. If he be the right man, he must be the heart and soul of the place, inspiring, co-ordinating, harmonising, and to a great extent he must work through his nursing staff. They must be in sympathy with his ideals, and they, too, must have the personalities to manage not only human bodies, but human beings, to understand not only medical cases, but living men.

